# Emergence of a novel high-level tigecycline resistance gene *tet*(X6) variant coexisting with *tet*(X2) and two *tet*(X) copies in a *Sphingobacterium* sp.

**DOI:** 10.1128/aac.01758-24

**Published:** 2025-03-10

**Authors:** Qiu Xu, Jie Hou, Stefan Schwarz, Jiyun Chai, Longhua Lin, Caiping Ma, Yao Zhu, Wanjiang Zhang

**Affiliations:** 1 State Key Laboratory for Animal Disease Control and Prevention, Harbin Veterinary Research Institute, Chinese Academy of Agricultural Sciences 687216 , Harbin, China; 2 Institute of Microbiology and Epizootics, Centre for Infection Medicine, School of Veterinary Medicine, Freie Universität Berlin 685237 , Berlin, Germany; 3 Veterinary Centre for Resistance Research (TZR), School of Veterinary Medicine, Freie Universität Berlin 9166 , Berlin, Germany; Universita degli studi di roma La Sapienza, Rome, Italy

**Keywords:** tigecycline, antimicrobial resistance, *tet*(X6) variant, *Sphingobacterium*

## LETTER

Tigecycline represents one of the last-resort drugs for the treatment of life-threatening infections caused by multi-drug-resistant (MDR) Gram-negative bacteria, especially carbapenem- and colistin-resistant Enterobacteriaceae. In 2019, the occurrence of mobile high-level tigecycline resistance genes, *tet*(X3) and *tet*(X4), in isolates from animals, humans, and the environment poses a significant threat to public health (1, 2). Subsequently, some other *tet*(X) variants, *tet*(X5)*–tet*(X22), were also identified in various species of bacteria (3). Among them, the genes *tet*(X4) and *tet*(X6) were reported to occur commonly (4). The *tet*(X6) gene was first identified on SXT/R391 integrative and conjugative element (ICE) ICE*Pgs*6Chn1 in a tigecycline-resistant *Proteus genomospecies* from retail pork in China (5). Similar to genes *tet*(X3) and *tet*(X4), the *tet*(X6) gene was able to confer high-level resistance to tigecycline, as well as the newly approved eravacycline and omadacycline. Until now, the *tet*(X6) gene has been identified in *Proteus*, *Acinetobacter*, and *Escherichia* (6). The genus *Sphingobacterium*, a member of *Bacteroidota*, was first described by Yabuuchi et al. in 1983 based on the presence of large amounts of sphingophospholipids in their cell membranes (7). Currently, the *Sphingobacterium* is regarded as an opportunistic pathogen associated with some human infections, such as bacteremia and peritonitis (8). In this study, we identified a novel chromosome-borne *tet*(X6) variant and its co-occurrence with *tet*(X2) and two *tet*(X) copies in a *Sphingobacterium* sp. BN32.

**Fig 1:**
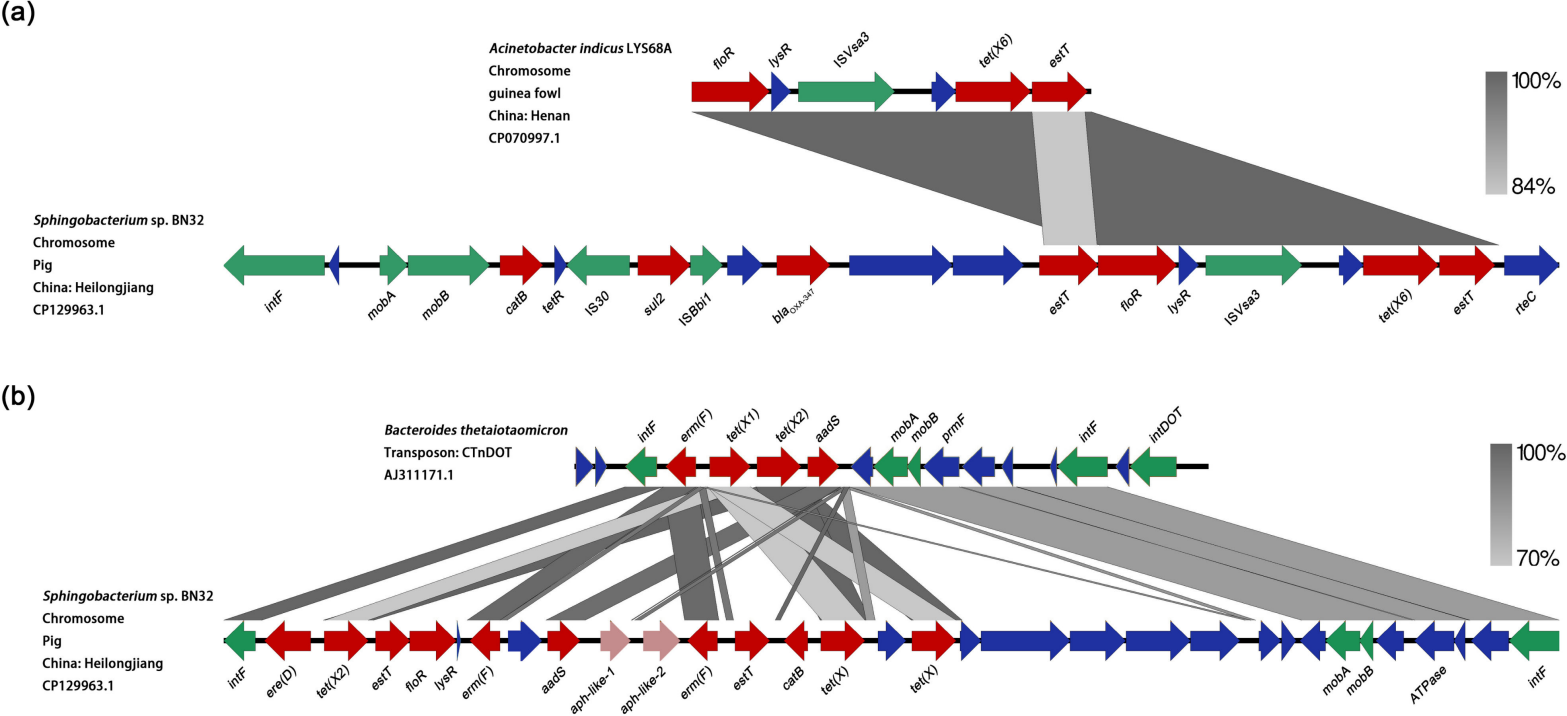
(a) Genetic environment of the *tet*(X6) variant in *Sphingobacterium* sp. BN32. (b) Genetic environment of two *tet*(X) and *tet*(X2) in *Sphingobacterium* sp. BN32. Arrows indicate the directions of transcription of the genes, and different genes are shown in different colors. Genes are color-coded as follows: green, mobilization; red, antimicrobial resistance; light red, putative antimicrobial resistance; blue, other proteins. Regions of homology are denoted by blue shading.

In October 2021, strain BN32 was isolated from the feces sample in Heilongjiang province, China, using a Columbia CNA blood agar plate containing tigecycline (2 mg/L). Species identification was conducted using 16S rRNA sequencing, and the result showed that strain BN32 was identified as *Sphingobacterium* sp. The genomic DNA of strain BN32 was extracted and subjected to whole genome sequencing (WGS) utilizing combined Illumina Hiseq (short reads) and Oxford Nanopore MinION (long reads) platforms. The *de novo* hybrid assembly was carried out using Unicycler v. 0.4.3, followed by genome annotation using both the RAST server (http://rast.nmpdr.org/) and BLAST (https://blast.ncbi.nlm.nih.gov/Blast.cgi). WGS revealed that strain BN32 harbored only a circular chromosome (4,373,748 bp). The whole genome sequences of strain BN32 have been deposited into the GenBank database under accession number CP129963. ResFinder analysis combining BLAST analysis indicated that strain BN32 carried multiple acquired antimicrobial resistance genes (ARGs), including *aadS*, *ere*(D), *estT* (four copies), *sul2*, *bla*_OXA-347_, *erm*(F) (two copies), *floR* (two copies), *catB* (two copies), *tet*(X) (two copies), *tet*(X2), and *tet*(X6). Phylogenetic analysis among *tet*(X) variants revealed that this *tet*(X6) gene was a novel variant (Fig. S1), which exhibited 97.63% nucleotide identity to the original *tet*(X6) (GenBank accession no. MN507533). To verify the function of the *tet*(X6) variant, the intact ORF of the *tet*(X6) variant, together with the predicted promoter, was amplified and ligated into the vector pUC19. The recombinant vector was then transferred chemically to *Escherichia coli* DH5α, followed by antimicrobial susceptibility testing. A 16- to 64-fold increase in the MICs of all tested tetracyclines was observed for the *E. coli* transformant. Among them, the MIC for tigecycline was a 32-fold increase compared with *E. coli* DH5α carrying the empty vector (from 0.125 to 4 mg/L), and this indicated that the novel *tet*(X6) variant was active against tetracyclines, including tigecycline. To the best of our knowledge, this is the first report of the *tet*(X6) gene in *Sphingobacterium*. In order to investigate the transferability of the *tet*(X6) variant, a conjugative transfer assay was performed using rifampin-resistant *E. coli* C600 as the recipient. Despite repeated attempts, transconjugants were not obtained. Genetic context analysis revealed that this *tet*(X6) variant was located in an MDR region. A *ca*. 6-kb core fragment harboring the *tet*(X6) variant exhibited >99% identity (99% coverage) to the corresponding regions of chromosomes or plasmids in various *Acinetobacter* isolates (Fig. 1a). This observation suggested that the *tet*(X6) variant possibly originated from *Acinetobacter* sp. In addition, two identical *tet*(X) genes exhibited 99.91% nucleotide identity to the prototype *tet*(X) gene (GenBank accession no. M37699), with one transversional point mutation (C-T) at position 1,077. Sequence analysis showed that similar to the *tet*(X6) variant, two *tet*(X) genes together with the *tet*(X2) gene were also located in a large MDR region. Sequence comparison suggested that this MDR region was located in a CTnDOT-like structure, which shared similarity with a conjugative transposon CTnDOT (GenBank accession no. AJ311171) previously described in *Bacteroides* (9) (Fig. 1b). However, the *intDOT* gene, which coded for a tyrosine recombinase that was responsible for catalyzing the integration and excision of CTnDOT (10), was absent in the BN32 strain. Of note, two putative aminoglycoside resistance genes, which we named *aph*-like-1 and *aph*-like-2, respectively, were also found in the MDR region (Fig. 1b). Gene annotation and comparisons revealed that both of them encoded aminoglycoside phosphotransferases, which implied that they may be two novel aminoglycoside resistance genes and their functions need further investigation.

To conclude, we identified the emergence of a novel *tet*(X6) variant, together with *tet*(X2) and two *tet*(X) genes, mapped on the chromosome by different MDR regions in a *Sphingobacterium* sp. As a reservoir of ARGs, it is necessary to continue monitoring *Sphingobacterium* sp. of different origins.

## SUPPLEMENTAL MATERIAL

Fig. S1 aac.01758-24-s0001.docx Phylogenetic analysis of amino acid sequences of different *tet*(X) variants.

ASM does not own the copyrights to Supplemental Material that may be linked to, or accessed through, an article. The authors have granted ASM a non-exclusive, world-wide license to publish the Supplemental Material files. Please contact the corresponding author directly for reuse.

## References

[1] He T, Wang R, Liu D, Walsh TR, Zhang R, Lv Y, Ke Y, Ji Q, Wei R, Liu Z, et al.. 2019. Emergence of plasmid-mediated high-level tigecycline resistance genes in animals and humans. Nat Microbiol 4:1450–1456. doi:10.1038/s41564-019-0445-2 31133751

[2] Sun J, Chen C, Cui CY, Zhang Y, Liu X, Cui ZH, Ma XY, Feng YJ, Fang LX, Lian XL, Zhang RM, Tang YZ, Zhang KX, Liu HM, Zhuang ZH, Zhou SD, Lv JN, Du H, Huang B, Yu FY, Mathema B, Kreiswirth BN, Liao XP, Chen L, Liu YH. 2019. Plasmid-encoded tet(X) genes that confer high-level tigecycline resistance in Escherichia coli. Nat Microbiol 4:1457–1464. doi:10.1038/s41564-019-0496-4 31235960 PMC6707864

[3] Lu XY, Zhang LF, Peng K, Wang QJ, Liu RQ, Wang ZQ, Li RC. 2023. Characterisation of a novel tigecycline resistance gene tet(X22) and its coexistence with bla_NDM-1_ in a Pseudomonas caeni isolate. Int J Antimicrob Agents 62:106961. doi:10.1016/j.ijantimicag.2023.106961 37666436

[4] Zheng XR, Zhu JH, Zhang J, Cai P, Sun YH, Chang MX, Fang LX, Sun J, Jiang HX. 2020. A novel plasmid-borne tet(X6) variant co-existing with bla_NDM-1_ and bla_OXA-58_ in a chicken Acinetobacter baumannii isolate. J Antimicrob Chemother 75:3397–3399. doi:10.1093/jac/dkaa342 32766775

[5] He DD, Wang LL, Zhao S, Liu L, Liu JH, Hu GZ, Pan YS. 2020. A novel tigecycline resistance gene, tet(X6), on an SXT/R391 integrative and conjugative element in a Proteus genomospecies 6 isolate of retail meat origin. J Antimicrob Chemother 75:1159–1164. doi:10.1093/jac/dkaa012 32016288

[6] Usui M, Fukuda A, Suzuki Y, Nakajima C, Tamura Y. 2022. Broad-host-range IncW plasmid harbouring tet(X) in Escherichia coli isolated from pigs in Japan. J Glob Antimicrob Resist 28:97–101. doi:10.1016/j.jgar.2021.12.012 34936926

[7] Yabuuchi E, Kaneko T, Yano I, Moss CW, Miyoshi N. 1983. Sphingobacterium gen. nov., Sphingobacterium spiritivorum comb. nov., Sphingobacterium multivorum comb. nov., Sphingobacterium mizutae sp. nov., and Flavobacterium indologenes sp. nov.: glucose-nonfermenting gram-negative rods in CDC groups IIK-2 and IIb. Int J Syst Bacteriol 33:580–598. doi:10.1099/00207713-33-3-580

[8] Zhang R, Dong N, Shen ZQ, Zeng Y, Lu JY, Liu CC, Zhou HW, Hu YY, Sun QL, Cheng QP, Shu LB, Cai JC, Chan EWC, Chen GX, Chen S. 2020. Epidemiological and phylogenetic analysis reveals Flavobacteriaceae as potential ancestral source of tigecycline resistance gene tet(X). Nat Commun 11:4648. doi:10.1038/s41467-020-18475-9 32938927 PMC7494873

[9] Whittle G, Hund BD, Shoemaker NB, Salyers AA. 2001. Characterization of the 13-kilobase ermF region of the bacteroides conjugative transposon CTnDOT. Appl Environ Microbiol 67:3488–3495. doi:10.1128/AEM.67.8.3488-3495.2001 11472924 PMC93048

[10] Wood MM, Gardner JF. 2015. The integration and excision of CTnDOT. Microbiol Spectr 3. doi:10.1128/microbiolspec.MDNA3-0020-2014 PMC4480416 26104696

