# Supplementary material for: Emergence of a novel high-level tigecycline resistance gene tet(X6) variant coexisting with tet(X2) and two tet(X) copies in a Sphingobacterium sp
Source: Antimicrob Agents Chemother. 2025 Mar 10;69(4):e01758-24. doi: 10.1128/aac.01758-24 (PMC11963530; doi:10.1128/aac.01758-24)
Supplement: Fig. S1 — Phylogenetic analysis of amino acid sequences of different tet(X) variants. [file aac.01758-24-s0001.docx]

**
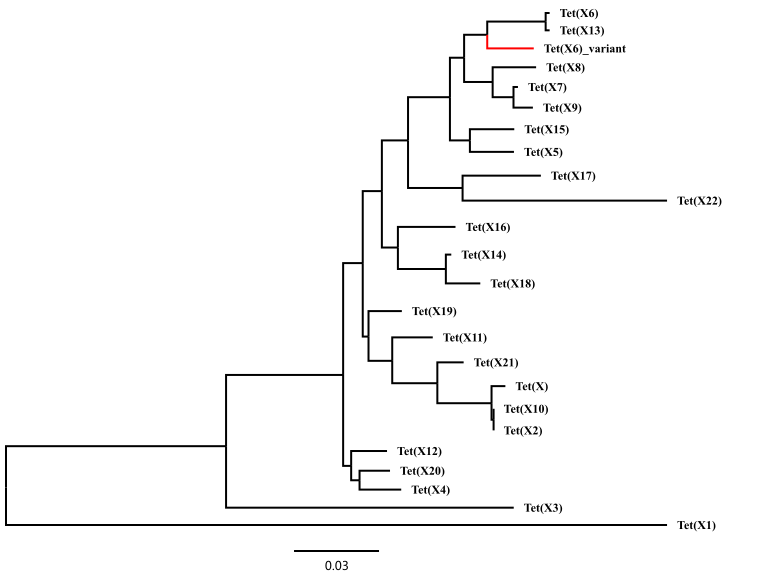
**

**Figure S1.** Phylogenetic analysis of amino acid sequences of different tet(X) variants. Tet(X6) variant labelled in red was identified in this study.
